# Omental torsion mimicking acute appendicitis in a 7-year-old boy: a case report

**DOI:** 10.1186/s13256-022-03515-3

**Published:** 2022-07-24

**Authors:** Elham Khalili, Mahdis Marashi, Majid Safarpanah, Saeedeh Majidi, Zahra Ghaeini Hesarooeyeh

**Affiliations:** 1 Student Research Committee, Faculty of Medicine, Hormozgan University of Medical Sciences, Bandar Abbas, Iran; 2 Universal Scientific Education and Research Network (USERN), Bandar Abbas, Hormozgan Iran; 3 Department of Radiology, Shahid Mohammadi Hospital, Hormozgan University of Medical Sciences, Bandar Abbas, Iran; 4 Department of Surgery, Shahid Mohammadi Hospital, Hormozgan University of Medical Sciences, Bandar Abbas, Iran; 5 Pediatric Surgery, Hormozgan University of Medical Science, Bandar Abbas, Iran

**Keywords:** Omentum, Torsion abnormalities, Acute abdomen, Case report

## Abstract

**Background:**

Torsion of the greater omentum is an uncommon cause of acute abdominal pain. Omental torsion can be divided into primary and secondary. Owing to its nonspecific abdominal pain, preoperative diagnosis is difficult. Ultrasound sonography and abdominal computed tomography scans do not have a major role in preoperative diagnosis. The definite diagnosis is usually made during operation.

**Case presentation:**

A 7-year-old Persian boy was referred with right lower quadrant pain associated with nausea and vomiting. Laboratory blood tests and urinalysis were requested that revealed normal values. Ultrasonography was performed, revealing inflammatory changes with mild free fluid collection in the interloop. The patient underwent an open appendectomy, confirming a normal appendix with omental torsion. Two days later, he was discharged home without postoperative complications.

**Conclusions:**

In patients with acute abdomen, particularly those with acute appendicitis symptoms, omental torsion should be considered in the differential diagnosis.

## Introduction

Omental torsion is a rare cause of acute abdomen [1] that results from omental rotation on its long axis, leading to a decreased blood supply and tissue infarction. Omental torsion mainly affects adults more than children, but its incidence has increased recently because of the widespread prevalence of obesity [2]. As Eitel had described for the first time in 1899, preoperative diagnosis of omental torsion is challenging because clinical symptoms and signs mimic other causes of abdominal pain, especially acute appendicitis. Omental torsion complications include intraperitoneal abscess, peritonitis, intraperitoneal bleeding, and rupture [3]. Surgical exploration is the gold-standard method for diagnosis [4]. In this case report, we describe a case of omental torsion in a 7-year-old boy presenting with symptoms of acute appendicitis.

## Case presentation

A 7-year-old Persian boy was referred to the emergency room with complaint of abdominal pain. His symptoms had started 2 days ago in the right lower quadrant and gradually increased, accompanied with nausea and vomiting.

He was fully vaccinated, had no significant family, social, or environmental history and had normal growth and development with no history of previous illness.

The child was not ill, and his vital signs included blood pressure of 111/720 mmHg, pulse rate of 75 beats per minute, respiratory rate of 17 breaths per minute, and temperature of 37 °C. Abdominal examination revealed right lower quadrant tenderness and rebound tenderness, while other parts of the abdomen had no pain or tenderness. There were no other findings on physical and neurological examination.

Laboratory tests such as complete blood count and liver function tests revealed normal values as follows: white blood cells 6.7 × 10^3^/µL (4.5–11 × 10^3^/µL) (neutrophils 58%, lymphocytes 31%); red blood cells 4.77 × 10^6^/µL (4.7–6.1 × 10^6^/µL); hemoglobin 10.1 g/dL (11.2–14.5 g/dL); hematocrit 33% (39–50%); mean corpuscular volume 69.2 fL (80–100 fL); red cell distribution width 17% (11.5–15%); platelets 294 × 10^3^/µL (150–450 × 10^3^/µL); aspartate aminotransferase 22 U/L (8–33 U/L); alanine transaminase 27 U/L (7–55 U/L).

**Fig. 1:**
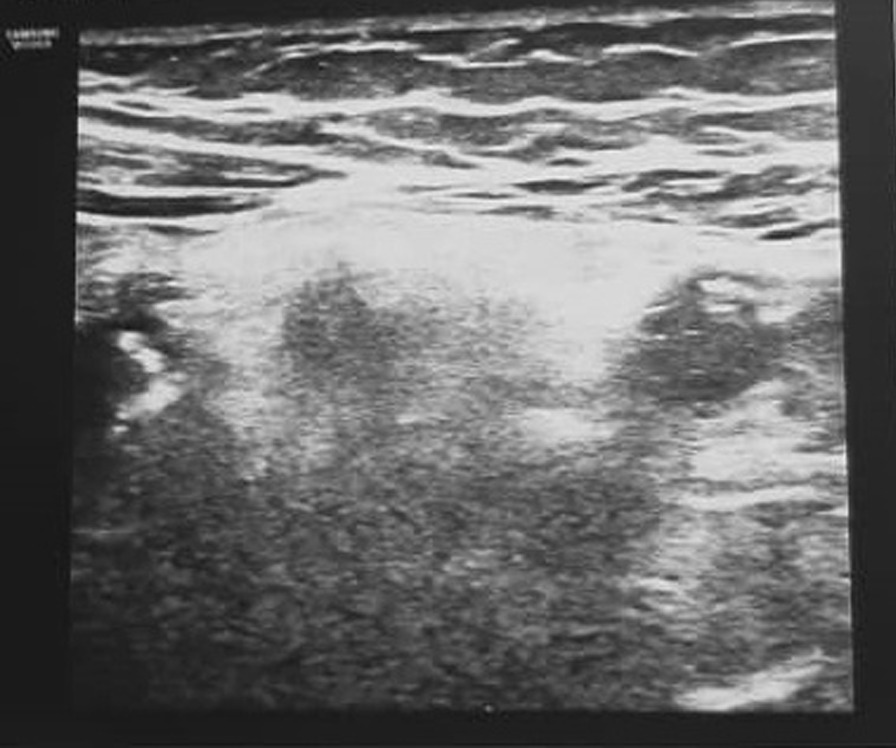
Ultrasonography showing hyperechogenicity of pericecal fat with mild free fluid in the interloop at the site of tenderness in the right lower quadrant

**Fig. 2:**
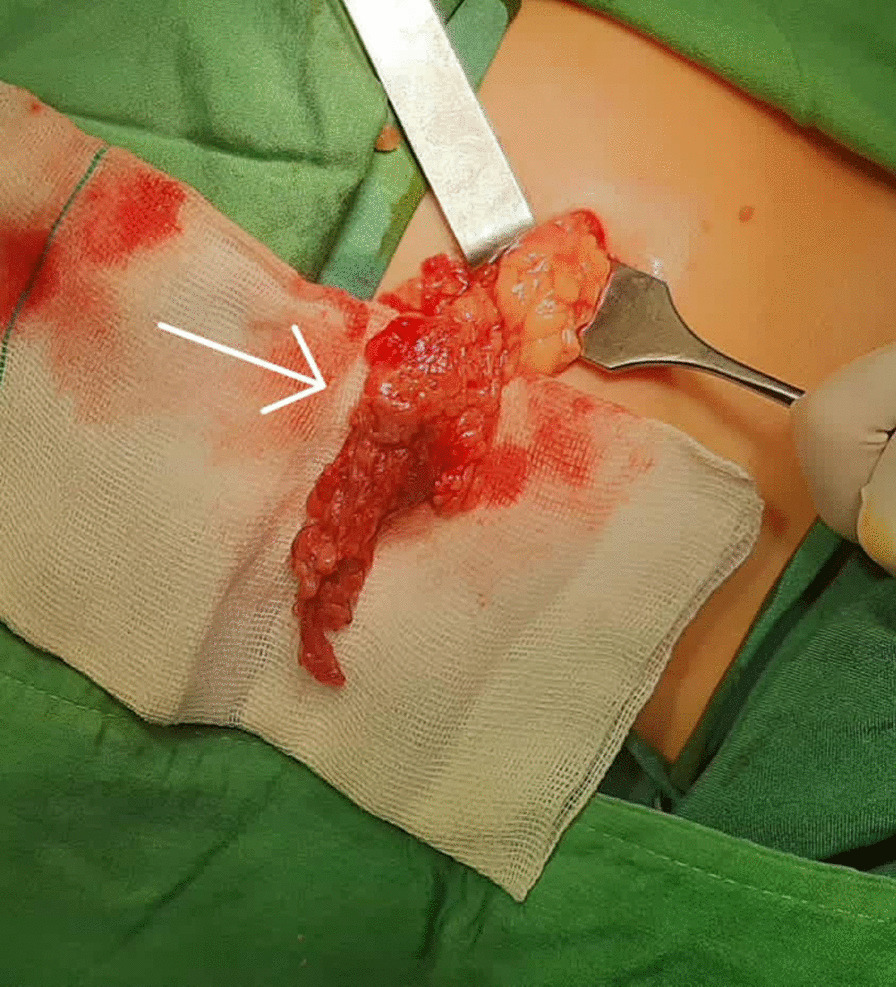
Intraoperative findings of omental torsion, before resection. The sudden color change is evident in the image (arrow)

Ultrasonography revealed enhanced pericecal fat echogenicity due to the inflammatory changes with mild free fluid collection in the interloop (Fig. 1). The patient underwent a diagnostic laparotomy with suspicious of acute appendicitis with a Lanz incision. The laparotomy, however, revealed a normal-appearing appendix, and greater omentum torsion was visually determined (Fig. 2). The incision was not extended, and laparotomic appendectomy and omentectomy were done through Lanz incision. The pathology report noted a creamy normal appendix, measuring 6 cm in length and 0.5 cm in greatest diameter. Omentum pathology was reported as fibroconnective tissue with congestion.

The patient was nil per os (NPO) after hospitalization.We hydrated the patient with stat 300 cc lactate Ringer and 600 cc dextrose 5% in 0.9% sodium chloride once every 8 hours. Antibiotic therapy include ceftriaxone 750 mg twice a day, metronidazole 300 mg every 8 hours, and analgesic [Apotel as needed (PRN)] was prescribed intravenously. After surgery, the patient’s symptoms were ameliorated and he was discharged from the hospital 2 days later. Oral medication including metronidazole, cefixime, and analgesic was prescribed.He had no complications on follow-up office visits after surgery for 6 months.

## Discussion and conclusions

The omentum is a four-layer sheet of the peritoneum that descends from the stomach and binds to the transverse colon. It is capable of sequestrating inflamed organs owing to its mobility [2] 4.

Omental torsion can be primary or secondary. Primary omental torsion is more prevalent in obese children and may occur in the absence of intraabdominal pathology. In contrast, secondary omental torsion, which is uncommon in children, is due to hernias, cyst or omental tumor, foreign body, adhesions, and postsurgical scarring. Risk factors for torsion include trauma, increased intraabdominal variations, pressure of fat deposits (particularly in obese patients), hyperperistalsis, omental anatomic variations, overeating, overexertion, and sudden changes in body position.

Increased omental weight due to fat deposits leads to decreased blood supply to the omentum in obese children and causes traction or torsion to the distal part. It occurs in 0.1–0.5% of children with presumptive appendicitis undergoing operations. Omental torsion occurs in adults more than children and is more common in males between 40 and 50 years (66%). With similar body weight, omental fat accumulation is higher in males compared with females of similar body weight, which may explain the higher prevalence in males [2] 5,6.

Pain is the main clinical presentation of omental torsion, and it can be located in the right upper or lower quadrants, depending on the omentum affected site. The pain is nonradiating and constant, with sudden onset [7, 8]. Most patients with omental torsion present only one episode of pain, and recurrent abdominal pain may suggest intermittent torsions [7]. Body movements may induce pain and can be associated with rebound and localized tenderness [6]. Patients can also present gastrointestinal symptoms, such as anorexia, nausea, and vomiting. Fifty percent of patients present leukocytosis and low-grade fever. A mass under examination may be palpable if a large omental segment is involved. The palpable mass can be found in 50% of patients [4, 9].

Clinical presentation simulates other acute abdominal conditions. Differential diagnoses include cholecystitis, acute appendicitis, cecal diverticulitis, abdominal wall hematoma, perforated duodenal ulcer, and intestinal obstruction. In women of reproductive age, ectopic pregnancy, ovarian cyst torsion, and salpingitis should also be included. In children, mesenteric adenitis and Meckel diverticulum should also be considered. Another diagnostic possibility is torsion of an accessory spleen when it presents, which is usually located inside the omentum [10]. The duration of omental torsion is longer than acute appendicitis, and patients are less systematically unwell [9].

In our experience, radiological studies, have usually been nonspecific. However, recent studies have outlined specific findings on ultrasonography, computed tomography (CT), and magnetic resonance imaging (MRI) [6]. The characteristic ultrasound finding in omental torsion is a noncompressible, hyperechoic oval mass adherent to the abdominal wall with a hypoechoic rim [3, 11]. CT scan is crucial in diagnosing omental torsion because it can differentiate omental torsion from appendicitis and acute cholecystitis by showing a normal, noninflamed appendix and gallbladder, respectively. Also, a paracolic abscess and bowel wall thickening on CT scan may suggest diverticulitis [12]. CT scan findings in omental torsion include a large cake-shaped or ovoid dense fatty mass with hyperattenuating streaks associated with the thick anterior abdominal wall. There is often a typical whirling pattern of the mesentery, with serosanguinous fluid accumulation within the peritoneum. However, these findings can also appear in other conditions, such as liposarcoma, lipoma, angiomyolipoma, mesenteric lipodystrophy, teratoma, pseudomyxoma peritonei, intestinal volvulus, and segmental infarction of the omentum [11] 13. In our case, clinical signs and examinations were conclusive of acute appendicitis, so there was no need for a CT scan. CT scan advantages outweigh ultrasonography on omental torsion evaluation when the mass can be identified in a specific location; in addition, ultrasonography is operator-dependent. Combining physical examination and real-time imaging may help focus on the maximal tenderness point by sonographic examination and less exposure to radiation [11]. MRI is also helpful, even with complications, such as abscess development or bleeding. However, the need for MRI is uncommon [7].

The treatment of omental torsion is controversial. Most patients recover with conservative treatment since imaging techniques can diagnose it early and lead to self-limiting and benign disease. Moreover, the total resolve of the inflammation, fibrosis, and retraction often occurs within 2 weeks [14] 15. However, some patients may require surgery because of an omental abscess, uncertain diagnosis, worsening of symptoms despite conservative treatment such as anti-inflammatory drugs, antibiotic therapy, and oral analgesics [14] 16. Surgical treatment includes laparoscopic resection of the affected segment of the omentum [17].

Furthermore, laparoscopic advantages are (1) facilitation of peritoneal aspiration and washing, (2) complete examination of the abdomen to confirm the diagnosis, and (3) reduction of the postoperative adhesion, wound-related complications, and postoperative pain [14].

In conclusion, omental torsion is a rare cause of acute abdomen. This case was treated successfully with omental resection. Most patients recover conservatively; however, because of the limitations in definitive diagnosis and prolonged hospital admission in conservative management, we suggest that surgical resection should be performed as soon as possible when the diagnosis is suspected.

## Data Availability

The datasets used during the current study are available from the corresponding author on reasonable request.

## Abbreviations

CT: Computed tomography
MRI: Magnetic resonance imaging
NPO: Nil per os
